# Retraction Note: Salinity-induced changes in the morphology and major mineral nutrient composition of purslane (*Portulaca oleracea* L.) accessions

**DOI:** 10.1186/s40659-023-00425-6

**Published:** 2023-03-20

**Authors:** Md. Amirul Alam, Abdul Shukor Juraimi, M. Y. Rafii, Azizah Abdul Hamid, Farzad Aslani, M. A. Hakim

**Affiliations:** 1 School of Agriculture Science and Biotechnology, Faculty of Bioresources and Food Industry, Universiti Sultan Zainal Abidin, Tembila Campus, 22200 Besut , Terengganu Malaysia; 2 Department of Crop Science, Faculty of Agriculture, Universiti Putra Malaysia, UPM Serdang, 43400 Serdang, Selangor, DE Malaysia; 3 Institute of Tropical Agriculture, Universiti Putra Malaysia, UPM Serdang, 43400 Serdang, Selangor, DE Malaysia; 4 Faculty of Food Science and Technology, Universiti Putra Malaysia, UPM Serdang, 43400 Serdang, Selangor, DE Malaysia

**Retraction: Biol Res (2016) 49:24** **https://doi.org/10.1186/s40659-016-0084-5**

The Editor-in-Chief has retracted this article because it contains substantial overlap with a previously published work by the same authors [1]. This article is therefore redundant.

Author Md. Amirul Alam disagrees with this retraction. The other authors have not responded to correspondence regarding this retraction.
